# Characterisation of the Metabolites of 1,8-Cineole Transferred into Human Milk: Concentrations and Ratio of Enantiomers

**DOI:** 10.3390/metabo3010047

**Published:** 2013-01-30

**Authors:** Frauke Kirsch, Andrea Buettner

**Affiliations:** 1 Department of Chemistry and Pharmacy, Food Chemistry, Emil Fischer Center, University of Erlangen-Nuremberg, 91052 Erlangen, Germany; E-Mail: frauke.kirsch@lmchemie.uni-erlangen.de; 2 Fraunhofer Institute for Process Engineering and Packaging IVV, 85354 Freising, Germany

**Keywords:** metabolism, human milk, gas-chromatography mass-spectrometry, cyclodextrin, enantiomers, chiral chromatography

## Abstract

1,8-Cineole is a widely distributed odorant that also shows physiological effects, but whose human metabolism has hitherto not been extensively investigated. The aim of the present study was, thus, to characterise the metabolites of 1,8-cineole, identified previously in human milk, after the oral intake of 100 mg of this substance. Special emphasis was placed on the enantiomeric composition of the metabolites since these data may provide important insights into potential biotransformation pathways, as well as potential biological activities of these substances, for example on the breastfed child. The volatile fraction of the human milk samples was therefore isolated via Solvent Assisted Flavour Evaporation (SAFE) and subjected to gas chromatography-mass spectrometry (GC-MS). The absolute concentrations of each metabolite were determined by matrix calibration with an internal standard, and the ratios of enantiomers were analysed on chiral capillaries. The concentrations varied over a broad range, from traces in the upper ng/kg region up to 40 µg/kg milk, with the exception of the main metabolite α2-hydroxy-1,8-cineole that showed concentrations of 100–250 µg/kg. Also, large inter- and intra-individual variations were recorded for the enantiomers, with nearly enantiomerically pure α2-hydroxy- and 3-oxo-1,8-cineole, while all other metabolites showed ratios of ~30:70 to 80:20.

## 1. Introduction

1,8-Cineole is a broadly distributed natural odorant with an eucalyptus-like smell that also gave the substance the common name eucalyptol. 1,8-Cineole belongs to the class of monoterpenes and is present in many herbs used in everyday cooking and in commercial foods, such as basil, rosemary, sage, cardamom, ginger, and peppermint [1,2,3]. The most important natural source is eucalyptus essential oil, more than 80% of which is 1,8-cineole [4,5].

1,8-Cineole is an interesting substance, not only because of its presence in food, but also, because it has several pharmaceutical properties and is used for human medicinal treatment. The broadest field of application for 1,8-cineole is in the therapy of severe pulmonary diseases such as asthma, where the compound exhibits mucolytic, bronchodilating, and anti-inflammatory properties [6,7,8,9]. Similar effects play a role in the treatment of acute sinusitis, where 1,8-cineole is the pharmacologically active agent in some non-prescription pharmaceuticals [10,11].

Metabolites can play an essential role for both desirable pharmacological effects as well as for undesired side effects of a substance. Thus, when evaluating the physiological impact of a certain compound, information about its potential biotransformation needs to be acquired. Although such studies are necessary for all pharmaceuticals in order to obtain final approval, and many metabolism studies have been conducted, for example, for environmental contaminants, food ingredients are commonly considered to be generally safe and thus have been studied in less detail, also with regards to metabolisation. Nevertheless, natural food ingredients are increasingly coming into focus in physiological research. Here, an interesting group of substances are terpenes [12,13], and especially monoterpenes, since they are widespread in many herbs and spices that are part of the human diet or are utilised in cosmetics or household products, via which dermal absorption or inhalation of volatile substances is possible. Terpenes are often biologically active, as they are synthesised in plants for functions such as attraction of insects for pollination, repelling herbivores, or as signal transducers (phytohormones) in the regulation of the plant metabolism [14]. Here, some common odorants and especially monoterpenes have indeed been shown to be critical from a toxicological point of view, often due to the formation of toxic metabolites [15,16]. Some examples are the allylalkoxybenzenes estragole, methyl eugenol, and safrole, which are carcinogenic in animal experiments and might therefore also be a risk to human health [17,18,19,20,21]. For 1,8-cineole, no such negative effects from animal experiments have been reported so far, but data are rather scarce. No carcinogenicity, genotoxicity, or reproductive or developmental toxicity has been reported up until now and subacute nephrotoxic and hepatotoxic effects in animal experiments appeared only after the application of high doses [3], in accordance with a rather high acute oral LD_50_ in rats of 2.5 g/kg bodyweight [22].

Physiological studies on terpenes, most specifically with regard to metabolic conversion in humans, are still few in number. While some early studies on human metabolism date back to the 1980s, this field of research has only started to grow notably in recent years. Some recent studies on the human metabolites of terpenes concern, for example, carvone [23,24,25], estragole [26], and 1,8-cineole [27,28,29].

The aim of the present study was to characterise the human metabolites of 1,8-cineole that had previously been identified in human milk after the oral intake of 100 mg encapsulated 1,8-cineole [30], most of which had not been found in other studies on the human metabolism of 1,8-cineole [27,28,29].

Sample extracts for gas chromatography - mass spectrometry (GC-MS) were prepared by solvent assisted flavour evaporation (SAFE), a gentle isolation method for volatiles [31]. The metabolites were quantified in the human milk samples via internal standard and matrix calibration, because absolute concentrations are crucial for evaluating the impact of each metabolite if, for example, the bioactive effects of the metabolites were to be addressed in future studies. Moreover, one might assume that human milk might show a totally different metabolite profile than the usually investigated plasma and urine [30]; this could be due to the unique physico-chemical properties of the milk matrix (oil-in-water emulsion, micelles), but also due to transfer processes, from blood to milk, which might lead to discrimination and accumulation effects [32]. Human milk is the most important nutritional source for breastfed newborns, thus knowledge of the concentrations of potentially transferred exogenous compounds in the milk is of utmost importance regarding the possible effects on breastfed children. Accordingly, the transfer of drugs into breast milk has long been the subject of many scientific studies [33,34,35], while the transfer of metabolites has attracted less attention. Since metabolism studies have hardly ever considered transfer or generation of metabolites in breast milk, it would be necessary to compare metabolite profiles in the different body fluids in order to be able to subsequently estimate values for breast milk, for example compared to the data obtained for plasma and urine.

Furthermore, regarding possible biological effects, chirality is a crucial point because, often, only one of two enantiomers is biologically active, or at least more potent than the other one [36,37,38]. Concerning pharmaceuticals, it is even discussed if enantiomerically pure preparations would be advantageous when comparing physiological benefits with potentially higher production costs [36]. Moreover, knowledge about the ratio of enantiomers of a chiral metabolite might also allow conclusions to be drawn about the respective metabolic pathways and can help characterise the relevant enzymes in terms of stereoselectivity. Thus, the ratio of enantiomers in the identified metabolites of 1,8-cineole was also determined in the present study via gas chromatographic analysis using different chiral β- and γ-cyclodextrin capillaries.

## 2. Results

### 2.1. Quantification of the Metabolites of 1,8-Cineole in Human Milk

#### 2.1.1. Concentration Ranges in Human Milk Samples

**Figure 1:**
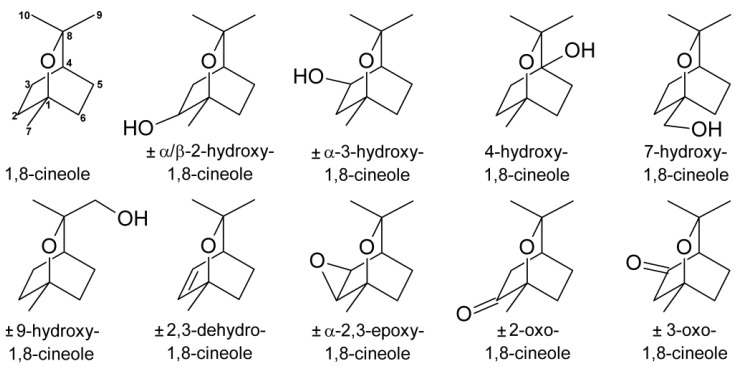
Metabolites of 1,8-cineol identified in human milk samples.

The chosen calibration method using spiked matrix samples and an internal standard, structurally similar to the analyte, yielded acceptable results for the quantification of ten metabolites of 1,8-cineole (Figure 1). An optimised calibration equation was established for each metabolite and concentration range, and recoveries of the calibration samples were found to range from 87% to 113%, with one exception of 120%. The linearity was tested with Mandel's fitting test and was considered acceptable for all except two calibration curves at significance levels of p ≤ 0.05. For the other two calibration curves, a quadratic equation would have given a better mathematic fit. However, visual inspection indicated that a linear fit was acceptable and, since no obvious reason for a quadratic correlation was found, the linear equation was used for quantitation of the samples. The correlation coefficients of the calibration equations were all higher than 0.9900. As one milk sample showed an exceptionally high content of α2-hydroxy-1,8-cineole, one calibration point at a ratio of ~1:20 was added to the calibration curve for this sample only. In this case, the calibration equation was optimised to give the best recovery at only the respective calibration point (100.1%) and the linearity and correlation coefficient were not considered.

Table 1, Table 2 report the quantification results for the ten metabolites 2,3-dehydro-, α2,3-epoxy-, α2-hydroxy-, β2-hydroxy-, α3-hydroxy-, 4-hydroxy-, 7-hydroxy-, 9-hydroxy-, 2-oxo- and 3-oxo-1,8-cineole in each analysed human milk sample. As mentioned in the experimental section, results for α3-hydroxy- and 3-oxo-1,8-cineole should be considered as “quantified as α2-hydroxy- and 2-oxo-1,8-cineole”, respectively, thus they are only estimates.

Additional information is given in the first column of Table 1 about the odour of the sample that was determined in a previous study [39]. Here, odourless samples (called “negative samples”) were also shown to contain only small amounts of 1,8-cineole in contrast to the samples with detectable eucalyptus-like odour (called “positive samples”), for which the transfer of 1,8-cineole into the human milk was more effective. As can be seen from the quantitative results for each metabolite in Table 1, Table 2, the “negative samples” in all cases also contained fewer metabolites than the “positive samples”. The averages for each group of metabolites were compared between these two sample groups using a Mann-Whitney U test. No significant difference could be detected for the metabolite content of 2-oxo-1,8-cineole (*p* < 0.05). On the contrary, the concentrations of 2,3-dehydro- and α2-hydroxy-1,8-cineole were, in both cases, significantly different between the two groups with *p* < 0.001. β2-Hydroxy-, α3-hydroxy- and 3-oxo-1,8-cineole showed significantly different concentrations between the two groups with *p* < 0.01. For α2,3-epoxy, 4-hydroxy-, 7-hydroxy- and 9-hydroxy-1,8-cineole the difference between the groups was significant with *p* < 0.05.

**Figure 2:**
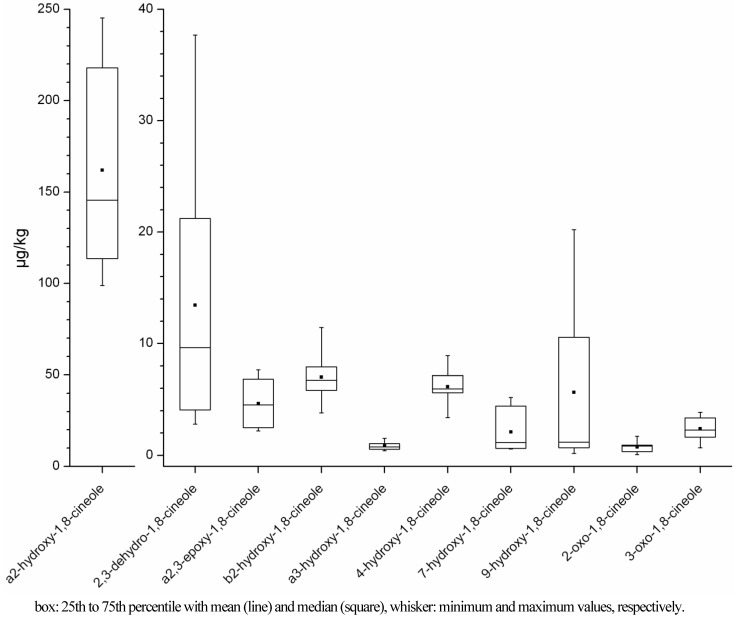
Boxplot of concentrations of metabolites of 1,8-cineole in human milk samples with eucalyptus-like odour.

For better visualisation of the concentrations of the different metabolites in the samples, a boxplot was generated for the seven “positive samples” (Figure 2). It can be clearly seen that α2-hydroxy-1,8-cineole is the metabolite with the highest concentration in human milk samples (99–233 µg/kg), and this was also the case when examining every sample separately. The concentration ranges of the other metabolites were about 10 to 1000 fold lower than for α2-hydroxy-1,8-cineole, *i.e.*, ranged from 0.1 to 38 µg/kg (Figure 2). From visual evaluation of the data it seems that 2,3-dehydro-1,8-cineole yielded the second highest concentration of all metabolites and that α3-hydroxy- and 2-oxo-1,8-cineole were present with the lowest concentrations. However, because of the small number of suitable samples, which arose from the limited availability of milk samples of sufficient volume (*i.e.*, due to lactation restraints), and because of the large variances between samples, none of these differences were statistically significant (*p* < 0.05).

**Table 1:** Absolute concentrations of metabolites of 1,8-cineole in human milk samples (single determinations).

Sample (odour^a^ yes/no)	1,8-cineole (µg/kg)^d^	2,3-dehydro-1,8-cineole (µg/kg)	a2,3-epoxy-1,8-cineole (µg/kg)	a2-hydroxy-1,8-cineole (µg/kg)	b2-hydroxy-1,8-cineole (µg/kg)	a3-hydroxy-1,8-cineole (µg/kg)
A-1a (no)	2.43 (<13.65)	0.28 (<1.26)	nd	0.65(<1.37)	nd	nd
A-1b (yes)	134.72	4.07	2.48	138.10	6.00	0.77 (<1.14)
B-1 (yes)	399.39	13.65	7.65	176.30	6.72	1.06 (<1.65)
B-2a (no)	0.98	0.07	nd	0.61	nd	nd
B-2b (yes)	70.97	2.80	2.18 (<2.45)	113.55	5.82	0.54 (<2.07)
B-2c (no)	14.82	0.57	0.66	29.25	1.71	0.05
C (no^b^)	13.56	0.73 (<0.88)	nd	7.68	0.33 (<0.96)	0.05 (<0.96)
D-1 (no)	5.75 (<10.59)	0.45 (<0.97)	1.35	15.08	1.42	0.03 (<1.06)
D-2 (yes)	226.09	5.13	4.13	135.98	7.35	0.41 (<1.92)
E-1 (no)	3.19 (<13.18)	0.19 (<1.21)	nd	2.02	nd	nd
E-2 (yes)	504.93	9.63	4.65	233.31	11.44	0.96 (<1.78)
F^c^ (no)	20.23 (<26.14)	0.59 (<2.40)	nd	3.16	nd	nd
G (yes)	194.46	21.24	4.52	98.83	3.80	0.72 (<3.51)
H (yes)	2089.54 (>1897.53)	37.69	6.82	206.09	7.90	1.53 (<1.96)

The samples marked as grey were beyond the calibration range. The calculated values are nevertheless reported as approximation. The concentration that is calculated with the last calibration point can be taken as an estimated maximum level, *i.e.* the samples contained less of the respective metabolite than this value, which is given in parentheses. Sample name code: each capital letter relates to one mother; the number relates to the measurement day (*i.e.* the occasion of intake of one Soledum® capsule) and the small letters designate samples from different times of the same measurement day. nd: not detected; ^a^ eucalyptus-like odour indicates positive transfer of ingested 1,8-cineole into human milk samples, as described in Kirsch *et al.* [39]; ^b^ very weak odour was detected but quantitative results [39] indicate that the smell was not caused by 1,8-cineole; ^c^ mother ingested two capsules; ^d^ values were taken from the original publication [39].

**Table 2:** Absolute concentrations of metabolites of 1,8-cineole in human milk samples (single determinations).

Sample (odour^a^ yes/no)	4-hydroxy-1,8-cineole (µg/kg)	7-hydroxy-1,8-cineole (µg/kg)	9-hydroxy-1,8-cineole (µg/kg)	2-oxo-1,8-cineole (µg/kg)	3-oxo-1,8-cineole (µg/kg)
A-1a (no)	nd	nd	nd	0.13 (<1.57)	0.01 (<1.57)
A-1b (yes)	5.96	0.56	0.46 (<1.08)	0.53 (<1.31)	1.80
B-1 (yes)	6.35	1.14	0.87 (<1.57)	0.91 (<1.89)	3.32
B-2a (no)	nd	0.21	0.18	nd	nd
B-2b (yes)	5.62	4.42	15.52	0.34 (<2.37)	1.47 (<2.37)
B-2c (no)	2.32	0.18	0.15	0.07	0.68
C (nob)	nd	nd	nd	nd	0.14 (<1.10)
D-1 (no)	2.24	nd	nd	nd	0.77 (<1.22)
D-2 (yes)	7.13	0.62	0.46 (<1.82)	0.85 (<2.20)	2.12 (<2.20)
E-1 (no)	nd	nd	nd	nd	nd
E-2 (yes)	8.92	5.19	16.95	1.71 (<2.03)	2.41
F^c^ (no)	3.62	nd	nd	2.71 (<3.00)	0.07 (<3.00)
G (yes)	3.39	0.84 (<1.06)	0.73 (<3.33)	nd	3.38 (<4.02)
H (yes)	5.66	1.87	1.83 (<1.86)	0.86 (<2.24)	3.85

For table notes please see Table 1.

For the “negative samples” too few quantitative values were obtained, thus no statistics could be applied. Nevertheless, the concentration of α2-hydroxy-1,8-cineole was also higher here than the concentrations of every other metabolite, except for one sample (sample F). While in the “positive samples” nearly every metabolite could be detected (with the only exception being 2-oxo-1,8-cineole in sample G), the “negative samples” (except sample B-2c) did not contain every metabolite at detectable amounts. Therefore it was not always the same metabolites that gave no signal, but different metabolites in different samples. Only α2-hydroxy- and 2,3-dehydro-1,8-cineole could be detected in every “negative sample”.

#### 2.1.2. Time-Dependency of 1,8-Cineole Metabolism

**Figure 3:**
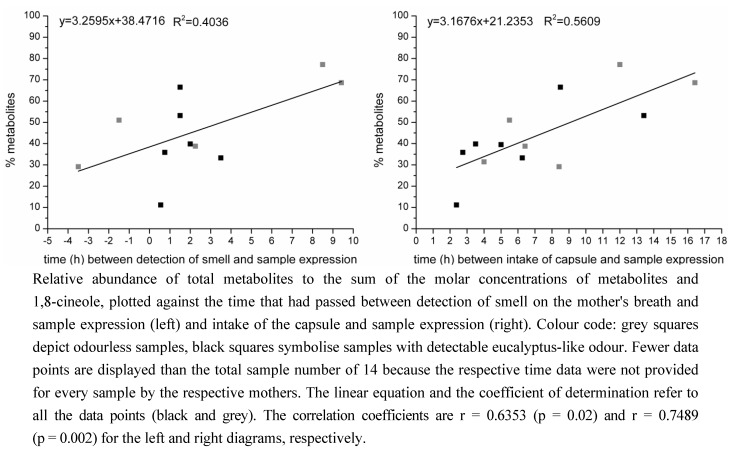
Time-dependency of metabolite generation.

Using the concentration of 1,8-cineole in the human milk samples under study [39] it was possible to calculate the relative molar abundances of metabolites to the “total molar concentrations” (total molar concentration as sum of the respective metabolites and 1,8-cineole). These values were correlated with the time that had passed between the intake of the capsule and the time of milk expression, and between the detection of the eucalyptus-like odour on the mother's breath and the time of sample expression, as depicted in Figure 3. Plotting the percentage of metabolites against the time between the detection of smell and sample expression indicated a linear correlation with a correlation coefficient of 0.6353 (*p* = 0.02, determined by T-test); plotting the data against the time between capsule intake and sample expression showed a correlation coefficient of 0.7489 (*p* = 0.002, determined by T-test) for an assumed linear correlation. Moreover, application of the Neumann trend test showed a significant trend (*p* < 0.05) for the correlation of the percentage of metabolites with the time between capsule intake and sample expression.

**Figure 4:**
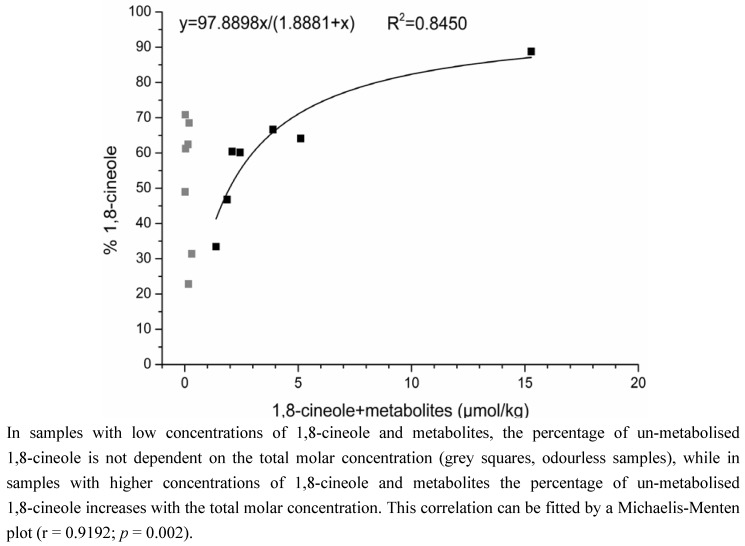
Correlation between the percentage of un-metabolised 1,8-cineole and the total molar concentration of 1,8-cineole and its metabolites.

Figure 4 depicts the relative molar abundance of un-metabolised 1,8-cineole to the total molar concentration, plotted against the total molar concentration. With increasing total molar concentration, the percentage of un-metabolised 1,8-cineole increased from 34% to 89% (*i.e.*, the percentage of metabolised 1,8-cineole decreased), resulting in a data distribution that can be plotted according to Michaelis-Menten, with a coefficient of determination of 0.8450 and a correlation coefficient of 0.9192 (p = 0.002, determined by T-test). It is noteworthy that this fit only applies to the samples with detectable odour, which also show high concentrations of 1,8-cineole [39]. The “negative”, odourless samples only contained small amounts of 1,8-cineole (and metabolites) and the impact of metabolism is here not dependent on the total molar concentration because the percentage of un-metabolised 1,8-cineole is distributed over a wide range from 23% to 71%.

### 2.2. Determination of the Ratios of Enantiomers

The separation of the chiral metabolites of 1,8-cineole was successfully achieved using different β- or γ-cyclodextrin-based stationary phases (Rt-bDEXsa, Rt-bDEXse, Rt-bDEXsm and Rt-yDEXsa) for gas chromatographic analysis. Table S1, in the supplementary material, lists the usable separation phases and the respective retention indices (determined according to van den Dool and Kratz [40]) for each pair of enantiomers. It was therefore decided to use the following capillaries for the analysis of the human milk samples: Rt-bDEXsm for separation of 2,3-dehydro-, α2,3-epoxy-, 2-oxo-, 3-oxo- and 9-hydroxy-1,8-cineole, and Rt-yDEXsa for separation of α2-hydroxy-, α3-hydroxy- and β2-hydroxy-1,8-cineole, under the GC conditions described in the experimental section.

The respective ratios and concentrations of enantiomers for each metabolite are listed in Table 3. The enantiomer eluting first on the respective GC capillary is named E1, the other E2. Comparison with the literature [41] allows the conclusion that E1 of α2-, β2-, α3- and 9-hydroxy-1,8-cineole as well as of 3-oxo-1,8-cineole is the (+)-enantiomer. Since there was no significant difference between the groups of samples with and without the eucalyptus-like odour (evaluated by Mann-Whitney U test, *p* ≤ 0.05), the results are given for the whole sample pool. The number of samples taken into consideration for the determination of the enantiomeric ratios differed from compound to compound due to the fact that not every metabolite could be detected in every sample. Moreover, some results had to be considered as outliers (tested for by Grubbs' test, *p* ≤ 0.01): In sample B-2a, E1 of 2,3-dehydro-1,8-cineole accounted for 100% because E2 was not detectable at all. In the same sample, E1 of α2-hydroxy-1,8-cineole was 40.5%. Samples C and B-2a showed 100% of E2 for α2,3-epoxy-1,8-cineole, and samples B-1, B-2b and B-2c contained 100% of E2 for 2-oxo-1,8-cineole. Even without applying statistics, comparison of these values with those listed in Table 3 clearly identifies these samples as outliers. These values were consequently not taken into account for calculation of the mean values and ranges reported in Table 3.

**Table 3:** Ratios of enantiomers and their absolute concentration for chiral metabolites of 1,8-cineole in human milk.

Metabolite	Percentage E1^a^ Mean (range)	Concentration E1^a^ (µg/kg) Mean (range)^c^	Percentage E2^b^ Mean (range)	Concentration E2^b^ (µg/kg) Mean (range)^c^	Number of Samples^d^
2,3-dehydro-1,8-cineole	52.8 (49.6–58.1)	4.08 (0.11–18.70)	47.2 (41.9–50.4)	3.98 (*0.08*–18.99)	12
a2,3-epoxy-1,8-cineole	33.9 (25.8–42.0)	1.29 (0.28–2.09)	66.1 (58.0–74.2)	2.74 (0.38–5.67)	8
a2-hydroxy-1,8-cineole	3.6 (0–7.8)	4.31 (0–15.99)	96.4 (92.2–100)	92.31 (1.91–219.65)	12
b2-hydroxy-1,8-cineole	40.1 (28.1–68.9)	2.01 (0.89–3.21)	59.9 (31.1–71.9)	3.79 (0.44–8.23)	9
a3-hydroxy-1,8-cineole	75.4 (68.5–80.0)	0.46 (0.02–1.16)	24.6 (20.0–31.5)	0.15 (*0.01*–*0.37*)	10
9-hydroxy-1,8-cineole	67.2 (50.4–81.9)	2.51 (0.09–8.65)	32.8 (18.1–49.6)	2.11 (*0.08*–8.30)	8
2-oxo-1,8-cineole	27.3 (21.2–31.5)	0.41 (0.24–0.78)	72.7 (68.5–78.7)	1.12 (*0.59*–*1.93*)	4
3-oxo-1,8-cineole	3.3 (0–5.0)	0.08 (0–0.19)	96.7 (95.0–100)	1.74 (*0.07*–3.65)	11

^a^ first enantiomer eluting on the respective chiral GC capillary; ^b^ second enantiomer eluting on the respective chiral GC capillary; ^c^ numbers in italics: samples were beyond the calibration range; the concentration was nevertheless calculated but has to be regarded as an approximation; ^d^ number of samples taken into consideration.

**Figure 5:**
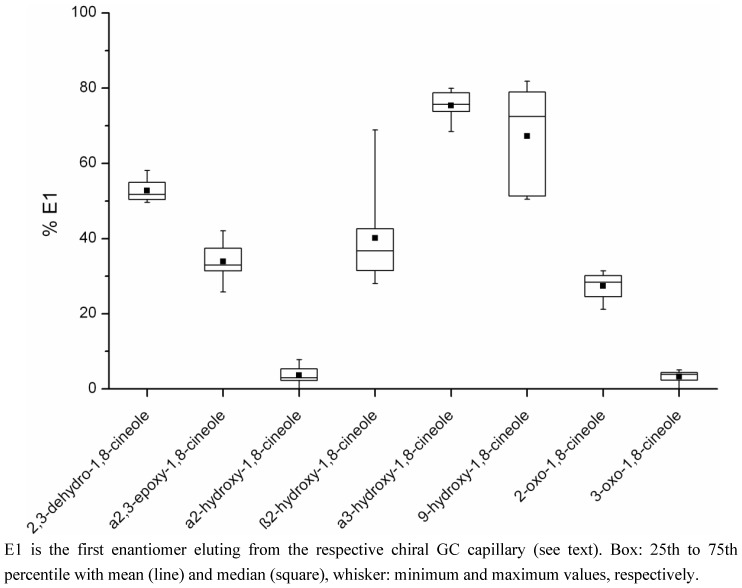
Boxplot of the percentage of enantiomer E1 for the chiral metabolites of 1,8-cineole detected in human milk samples.

For better visualisation of the results and the scatter of the data, percentage E1 for every chiral metabolite is depicted as a boxplot in Figure 5. The results for each metabolite were quite different: α2-Hydroxy- and 3-oxo-1,8-cineole both consisted of under 10% E1 and data were distributed over a narrow range. For 2,3-dehydro-, α2,3-epoxy-, 2-oxo- and β2-hydroxy-1,8-cineole, E1 was in the 20% to 60% range; thereby β2-hydroxy-1,8-cineole showed quite a broad data distribution over the whole range while the scattering of the values of the other metabolites was more narrow. Also for 9-hydroxy-1,8-cineole, percentage E1 covered a large range, from about 50 to 80%. Finally, α3-hydroxy-1,8-cineole exhibited a rather narrow data scatter of around 70% to 80% E1.

The individual data for each milk sample (not shown) allow another interesting observation concerning β2-hydroxy-1,8-cineole to be made. Although for all the other metabolites one of the enantiomers, E1 or E2, showed consistently the higher concentration in every sample irrespective of the variances between samples, in the case of β2-hydroxy-1,8-cineole some samples were higher in E1, but others also in E2. Even two samples from the same mother often differed, namely E1 was the predominant enantiomer in one sample, yet in the other one it was E2. Also for 9-hydroxy-1,8-cineole, with the values distributed over a very broad range, the three samples obtained from the same mother (samples B-2a, B-2b, B-2c) did not show the same ratio: In one sample, it was about 80% E1, in the two other samples it was only about 50%.

## 3. Discussion

### 3.1. Quantification of the Metabolites of 1,8-Cineole in Human Milk

#### 3.1.1. Quantification Method Using Matrix Calibration

The use of stable isotope dilution assays is state of the art in flavour analysis. However, isotopically-labelled standards are expensive or, mostly, not commercially available, and syntheses with the required high purity are time-consuming and far from trivial. For non-routine analyses, as in the present study with several different analytes but only a limited number of samples, the effort of providing isotopically-labelled analogues for every analyte is not justifiable. Nevertheless, the applied quantification method requires the use of internal standards and even better labelled analogues of the analytes. Although being the optimal method for isolation of volatiles from complex matrices, sample preparation via SAFE can lead to relatively large absolute losses of analytes, as experienced in former studies and also described in the original publication introducing the technique [31]. Extensive concentration of large solvent volumes bears another risk for loss of analytes. Moreover, the composition of the sample, especially the fat content, can affect the release of the volatiles. The impact of these effects depends on the individual sample, making compensation with a generally applied correction factor impossible.

Usage of internal standards can adjust for losses, but if standard and analyte are likely not to experience exactly the same losses, possible discrimination effects should be taken into account by evaluating the response between internal standard and analyte under the conditions of sample preparation, that is, to establish a matrix calibration. However it should be mentioned that the matrix actually used for calibration in the present case might have been different to some extent from that of the samples, especially since the fat content of human milk samples generally varies over a wide range [42]. Nevertheless the applied method was the best approximation possible. Isotopically-labelled 1,8-cineole was chosen as an internal standard due to its high structural similarity to the metabolite molecules.

Due to the mentioned variations in sample preparation, each calibration level was prepared in triplicate. For each metabolite, the calibration equation was optimised as described in the methods section to give the best recovery rate when recalculating the concentration of the employed calibration samples with this equation. With resulting recoveries from 87% to 113%, these calibration equations are comparable to those usually established for the quantification of 1,8-cineole, with an isotopologue of the analyte as an internal standard and in solvent instead of the matrix [39], where the recoveries for the calibration ranged from 85% to 105% (unpublished data). Moreover, the correlation coefficients of the calibration curves were quite good (R^2^ > 0.9900) and nearly all of them were linear when tested by Mandel`s fitting test. Thus, although the described quantification method was not perfect it yielded acceptable results.

#### 3.1.2. Time-Dependency of 1,8-Cineole Metabolism

It could have been anticipated that samples with higher concentration of 1,8-cineole also showed higher concentrations of metabolites, so that the percentage of metabolites from the total molar concentration of metabolites and 1,8-cineole would have been nearly the same for every sample. However, this was not the case; further, the diagrams displayed in Figure 3 might be interpreted as showing a time-dependency of metabolite generation. The correlation coefficients are rather low due to the small sample number, but the correlation is significant and Neumann`s trend test was also significant, at least for the correlation between percentage of metabolites and the time between capsule intake and sample expression. A possible reason for such a time-dependency could be that the longer the parent molecule 1,8-cineole had been inside the maternal body, the more of it could have been converted to metabolites by the respective enzymes. This trend might be counteracted by the saturation of the responsible enzymes as discussed below, which might cause the scattering around the linear regression line. Since the limited sample number might bias the correlation, linearity has to be treated with caution here. It might as well be, for example, a Michaelis-Menten-like correlation that reaches a plateau due to enzyme saturation, which might not be seen here because the samples are still in the ascending part of the respective curve. However with the given number of samples a linear correlation is the one that gives the best fitting regression.

Interestingly, the linear relationship is better when correlating the percentage of metabolites with the time that had passed between the intake of the capsule and sample expression than with the time passed between the detection of the eucalyptus-like smell on the breath and the expression of the sample. Since the detection of the smell on the breath was used to determine the best time for sample expression, signifying the maximum absorption of 1,8-cineole, it should be expected that this time showed the better correlation. A possible explanation for this inverse effect might be that the time of capsule intake could be definitely determined, while the detection of the smell on the own breath was difficult for many participants and is therefore clouded by uncertainty. The potentially resulting biased time data might compensate the effect of better correlation.

It is also noticeable that in the case of comparison of the percentage of metabolites with the time that passed between detection of smell and sample expression (Figure 3, left), the time-dependency seems to be caused only by the samples without detectable odour, *i.e.*, with low amounts of 1,8-cineole and metabolites. A possible explanation might be that the time data for the detection of the smell was, by chance, more accurate in these cases than for the other samples. From the right diagram in Figure 3 it can be seen that here both the “positive” and “negative” samples show a slight time-dependency. The fact that this time-dependency can already be seen when correlating the percentage of metabolites with the time that passed between capsule intake and sample expression indicates that metabolic processes could, to some extent, take place even before the detection of the eucalyptus-like odour on the breath indicated the maximum absorption of 1,8-cineole.

As already mentioned, the positive correlation of the percentage of metabolites and the time that the parent molecule 1,8-cineole spent inside the maternal body might be counteracted by a possible effect of enzyme saturation. During a normal, balanced diet and lifestyle, the usual uptake is probably rather low so the enzyme saturation is not reached. However, if high amounts, like in the present study, are taken up it is conceivable that the higher the concentration of 1,8-cineole, the lower the percentage of metabolised 1,8-cineole, because there is only a limited number of enzyme molecules and thus the reaction speed can only be increased up to a certain point. The present data (Figure 4) indeed indicate that such an effect of enzyme saturation did take place. The correlation of the Michaelis-Menten plot is not particularly good since, also, the number of samples is rather small; nevertheless, the correlation is significant and the respective trend is obvious. Especially when comparing the data points of the odourless samples (gray squares in Figure 4) with those of the samples with detectable odour and thus with higher concentrations of 1,8-cineole (black squares in Figure 4), it is noticeable that the latter exhibit a correlation between total molar concentration and percentage of un-metabolised 1,8-cineole. In contrast to that, data points of the odourless samples are scattered over a wide percentage range (23% to 71%) while lying in a very small range of total concentration (0.01–0.31 µmol/kg).

Finally, from the results displayed in both Figure 3 and Figure 4, the overall conclusions can be drawn that if only small amounts of 1,8-cineole were present in the samples, predominantly the available time for metabolic reactions (*i.e.*, the time the parent molecule spent inside the maternal body before samples expression) played a role, also because the enzymes were still far from reaching saturation. However, if higher amounts of 1,8-cineole were present, enzyme kinetics became more important, being then the primary factor influencing the percentage of metabolised 1,8-cineole.

### 3.2. Possible Influences on the Enantiomeric Ratios of the Metabolites

The enantiomeric ratios for each metabolite are summed up in Table 3. Besides the percentage values, the absolute concentrations that were calculated from the concentrations of the metabolites reported in Table 1, Table 2 are given. Since some of the concentrations were only estimations because they lay below the calibration range, or could not be determined with an isotopologue as an internal standard (as in the case of α3-hydroxy- and 3-oxo-1,8-cineole), the respective concentrations of each single metabolite, and thus the reported ranges in Table 3, likewise have to be considered as estimations. However, the percentage values are not influenced by these shortcomings. Nevertheless, the results cannot be generalised due to the limited number of data, especially for 2-oxo-1,8-cineole, which could be detected in only four of the samples.

The large inter- and intra-individual variances of the present results can be explained with natural variances in biological samples. While absolute concentrations can vary due to individually different absorption rates [37] and different metabolic activities due to polymorphisms [43,44,45], or induction, or inhibition of certain enzymes [36,38,46], one might expect that the ratio of enantiomers would always remain constant. But since different enzymes may be involved in the metabolism, several scenarios are conceivable that might lead to great variations. Thus, the two enantiomers of one pair could be generated by two different enzymes, and induction and inhibition effects could further affect both enzymes differently, resulting in different enantiomeric ratios. Such effects could be caused by various substances from the diet and total lifestyle of the participating mothers and would not have been possible to control without intensive supervision of the participating subjects. Induction and inhibition could also have a great influence if the same metabolic reaction would lead to both enantiomers but was catalysed by more than one enzyme at the same time. Moreover, most enzymes show substrate and/or product stereo-selectivity [37,38,47], and many metabolites might additionally be connected to each other (for example 2-hydroxy- and 2-oxo-1,8-cineole). Accordingly, different enantiomeric ratios of one primary metabolite might further influence the enantiomeric ratio of the resulting product metabolite. The situation becomes even more complicated if one also assumes that “back reactions” might occur, *i.e.*, one metabolite that was generated from another might be converted to its precursor again (for example 2-hydroxy- → 2-oxo- → 2-hydroxy-1,8-cineole). The involvement of different enzymes that might be up or down-regulated, according to the individual metabolic status, would thus lead to a broad spectrum of possible resulting enantiomeric ratios. 

Possible enzymatic reactions establishing relationships between the different metabolites might be the following: A classic detoxifying reaction is the epoxidation of a double bound [48] and accordingly 2,3-dehydro-1,8-cineole could be converted into α2,3-epoxy-1,8-cineole. 2,3-Dehydro-1,8-cineole itself might be generated by dehydrogenation of 1,8-cineole or dehydration of α2-hydroxy-, β2-hydroxy- or α3-hydroxy-1,8-cineole [48]. The 2- and 3-hydroxy-cineoles could be further oxidised to the respective oxo-cineoles [49] and a back-reaction might also be conceivable. Generally, many human enzymes are capable of catalysing the reaction of a carbonyl- to a hydroxyl-function (e.g. aldo-keto-reductases, carbonyl-reductases) [49,50], but focussed studies with molecules comparable to oxo-1,8-cineoles have, as of yet, not been accomplished. However, some bacterial enzymes have been reported to be responsible for very similar reactions such as the reduction of camphor to borneol [51], and human carbonyl reductases have such a broad substrate specificity [52] that it is well conceivable that such a back-reaction of oxo- to hydroxy- 1,8-cineole might take place, as also proposed by Ishida *et al.* for thujone and carvone [53].

Another aspect that has to be considered here is the transfer into the breast milk. Active absorption and transfer processes may also discriminate between enantiomers [37] and if the metabolites are not formed in the milk but are derived from the maternal blood, such an effect could contribute to the stereospecific metabolism profile. Protein binding in blood plasma has also been observed to influence stereo-selectivity concerning distribution of pharmaceuticals [37,47], and is conceivable in milk, too. This might influence the observed enantiomeric ratios but would moreover lead to an underestimation of concentration because these bound analytes would be lost during sample preparation.

Some curious results might only be explained by such complex interactions of different enzymes and different individual metabolic activities caused by polymorphisms, inhibition, and induction effects. Thus, α2-hydroxy- and 3-oxo-1,8-cineole seem to be rather enatiomerically pure, while α3-hydroxy- and 2-oxo-1,8-cineole show ratios between 40:60 and 30:70. This is the opposite of what would be expected if the ratios were conserved during the conversion. The enantiomeric distribution of α2-hydroxy-1,8-cineole is neither similar to that of the respective β-isomer, nor to that of the α3-hydroxy-1,8-cineole, indicating that they were not generated by the same enzyme if 1,8-cineole was the direct precursor. Moreover, β2-hydroxy-1,8-cineole showed the highest variation in enantiomeric ratio of all metabolites because the values for each enantiomer were not only scattered over a broad range, but some samples were higher in E1, while others were higher in E2. In contrast, for all other metabolites it was the same enantiomer that made up the major component in every sample. Only further experiments at the enzyme level could help to elucidate possible reaction pathways and connections between the single metabolites.

### 3.3. Comparison with Other Studies on Human 1,8-Cineole Metabolites

α2-Hydroxy-1,8-cineole was the major metabolite of 1,8-cineole in human milk, with at least 10-fold higher concentrations than other metabolites (Figure 2), which is in agreement with other studies on the metabolism of 1,8-cineole in humans using human liver microsomes [27,28,54,55]. Duisken *et al.* confirmed these results by additional analyses of human urine after volunteers took a 1,8-cineole-containing cold medication. The metabolites α2- and α3-hydroxy-1,8-cineole were detected, with α2-hydroxy-1,8-cineole again being the main metabolite. A human study by Horst *et al.* [29] analysed the urine and plasma of one volunteer who ingested 1,8-cineole by drinking sage tea, resulting in an overall intake of 1.02 mg 1,8-cineole. These authors found the metabolites α2-and 9-hydroxy-1,8-cineole in plasma at concentrations of 14.62 and 5.61 µg/L, respectively, and α3-and 7-hydroxy-1,8-cineole in amounts below the limit of quantitation. No absolute concentrations were reported for urine, but α2-hydroxy-1,8-cineole was the main metabolite, followed by 9-hydroxy-1,8-cineole. Another very recent study conducted by Schaffarczyk *et al.* [41] also analysed metabolites of 1,8-cineole in human urine after intake of the same amount 1,8-cineole (100 mg in one Soledum® capsule) as used in the present study. The metabolites were not absolutely quantified in this study but the authors could confirm the results by Horst and Rychlik [29], that α2-hydroxy-1,8-cineole was the major metabolite, followed in decreasing order by 9-, α3-, and 7-hydoxy-1,8-cineole. The same authors also identified the metabolites β2-hydoxy-, 4-hydroxy- and 3-oxo-1,8-cineole for the first time in human urine, matching the situation in human milk presented here.

Schaffarczyk *et al.* also reported the ratio of respective enantiomers identified in human urine [41]. Surprisingly, the enantiomeric ratios that could be observed in the urine samples were quite to the contrary of the results of the present study in human milk. While E2 dominated α2-hydroxy- and 3-oxo-1,8-cineole in human milk, urine samples exhibited the opposite. In both matrices, 3-oxo-1,8-cineole was nearly enantiomerically pure, but while this was also the case for α2-hydroxy-1,8-cineole in human milk, this metabolite was closer to the racemic state in the urine samples, with an average enantiomeric ratio of 38:62. 9-Hydroxy-1,8-cineole clearly showed a higher percentage of E1 in human milk, but a higher percentage of E2 in human urine. The same trend in both sample types only occurred for α3-1,8-cineole, with E1 being the predominant enantiomer. For β2-hydroxy-1,8-cineole the results in human milk were highly variable, with no clear preference for either enantiomers, while in urine the E1 molecule was present in excess.

There is a whole range of possible explanations for these discrepancies between the enantiomeric ratios in human milk and urine. One reason might be that the metabolites found in milk and urine do not originate from the same pool of metabolites in the bloodstream, but were generated at different locations. While there is no active metabolism in urine and the metabolites found in the urine samples were surely excreted from the blood, or generated within the kidney, it might well be that there is some respective enzymatic activity in human milk, as already discussed in a previous publication [30]. Moreover, for exchange between blood and milk or urine to occur, substances have to pass cells of many different types whose enzymatic equipment could also contribute to metabolic processes. Next, it might be conceivable that, although in the very first moment after the metabolites have been transferred into the different body fluids the profiles are the same, different conversion reactions involving (as discussed above) different enzymes, and enzymes with different selectivities, respectively, take place to modify the profiles. Another point already discussed above is the complex transfer processes finally leading to the presence of the metabolites in human milk or urine: Discrimination of one enantiomer during transfer might seriously alter the enantiomeric ratio. Moreover, especially important for the transfer into urine, the need for a conjugation to a more polar molecule before excretion is another critical point at which discrimination between enantiomers might be accomplished by the conjugating enzymes. Examples for a stereo-selective conjugation are the glucuronidation of morphine, where the (+)-enantiomer is preferred [56] and that of 2-phenylpropionic acid, where the molecule in the R-configuration is more readily glucuronidated [57].

When discussing the results of the present study in comparison to those of Schaffarczyk *et al.* [41] it is also a very remarkable difference that the metabolites 2,3-dehydro-, α2,3-epoxy- and 2-oxo-1,8-cineole could not be found in human urine, while they were present in human milk; especially 2,3-dehydro-1,8-cineole was quantified in amounts that were not negligible. A previous assumption, before the study of Schaffarczyk *et al.* [41] was available, was that these metabolites were due to the high applied dose of 1,8-cineole. The outcome of the urine study by Schaffarczyk *et al.* [41] disproved this theory, thus the reason must rather be a general difference between the metabolite profiles in these two body fluids. The results of the two studies particularly indicate that the different polarities of the matrices play a crucial role, since the metabolites 2,3-dehydro-, α2,3-epoxy- and 2-oxo-1,8-cineole are rather lipophilic in comparison to the other identified metabolites of 1,8-cineole and might thus be more easily soluble in the hydrophobic phase of milk than in the aqueous urine.

## 4. Experimental Section

### 4.1. Chemicals/Materials

Deuterium-labelled ^2^H_3_-1,8-cineole was supplied by aromaLAB AG (Munich, Germany). 2-Hydroxy-1,8-cineole, 7-hydroxy-1,8-cineole and 9-hydroxy-1,8-cineole were synthesised as described by Horst and Rychlik [29]. α3-Hydroxy-1,8-cineole was a generous gift from C. J. Wallis and R. M. Carman (University of Queensland, Brisbane, Australia). 2,3-Dehydro-1,8-cineole, α2,3-epoxy-1,8-cineole, 2-oxo-1,8-cineole, 3-oxo-1,8-cineole, β2-hydroxy-1,8-cineole and 4-hydroxy-1,8-cineole were synthesised as described by Kirsch *et al.* [30]. Unlabelled 1,8-cineole, E-2-decenal and anhydrous Na_2_SO_4_ were purchased from Aldrich (Steinheim, Germany) and dichloromethane was obtained from Acros (Geel, Belgium).

### 4.2. Human Milk Samples

Human milk samples were provided voluntarily by breastfeeding mothers after intake of the non-prescription pharmaceutical Soledum® (Klosterfrau Healthcare Group, Cologne, Germany). Before the beginning of the study, the participants were informed verbally, as well as in written form, about the study procedure and purpose and they were told that withdrawing from the study was possible at any time without giving any reason. All volunteers gave written consent to the analysis of their milk samples and anonymous publication of the resulting data. The Ethical Committee of the Medical Faculty, University Erlangen-Nuremberg, approved the experimental procedures. Each participant was handed a copy of the Soldeum® package instructions and was advised to read it before capsule intake. They were also advised to withdraw from the study if any negative reactions such as medication allergies or interactions with other drugs they were taking were to be expected. Additionally, the volunteers were informed verbally about potential effects of the pharmaceutical preparation on breastfeeding, *i.e.* the risk of an altered milk flavour and rejection of the milk by the nursling. Accordingly, if this would happen, the mothers were recommended to feed their babies with stored human milk or formula milk products.

The milk donors were between the ages of 29 and 40 years (mean 34 years). During their participation in the study, their breast milk production had to be normal and in excess of their infants' needs, with no breast infections and no overall medical complaints present. Five participants were primiparous, three were multiparous and the breast milk samples were from 19 weeks to 19 months postpartum. For sampling, either electrical or mechanical breast pumps or manual expression techniques were applied, resulting in sample volumes between 10 and 100 mL. The samples were immediately subjected to sample preparation after a maximum storage time of five hours at 7 °C or were stored in a freezer at −20 °C for up to five days.

The participating volunteers were asked to ingest one Soledum® capsule, containing 100 mg 1,8-cineole, and to wait until they themselves or another person began to notice the eucalyptus-like odour on their breath. Only then a milk sample should be expressed, within the next two to four hours, because previous studies [39] showed that the transfer of 1,8-cineole into human milk coincided with the first signs of the eucalyptus-like odour in the breath. The donors were instructed to keep notes on the dates of capsule intake, first eucalyptus-like odour on their breath and exact sampling time. Unfortunately it was difficult for some mothers to detect the smell on their breath so they expressed the samples after some time without having noticed the smell, or reported having been unsure about the smell. This resulted in several samples that did not show the typical eucalyptus-like odour. Some of the donors repeated the whole experiment and some expressed more than one sample after the capsule intake, resulting in 14 samples in total, from 8 different mothers.

### 4.3. Sample Preparation for GC Analysis by Solvent Assisted Flavour Evaporation

Extracts containing the volatile fraction of the human milk samples were prepared by Solvent Assisted Flavour Evaporation (SAFE) [31] after addition of deuterated 1,8-cineole as internal standard (solution in dichloromethane), followed by 10 min of stirring, then another addition of dichloromethane (50% v/v), and 30 min of stirring. The resulting stable phase was distilled in the SAFE-apparatus at 50 °C and approximately 1x10^−4^ mbar. The volatile fraction of the samples was frozen with liquid nitrogen. After thawing, it consisted of two phases, formed by the dichloromethane and by the water out of the milk. The water phase was extracted three times with dichloromethane, and the solvent phases were combined and dried over anhydrous Na_2_SO_4_. Finally the sample extracts were concentrated by microdistillation according to Bemelmans [58] at 50 °C to a total volume of 50 to 200 μL. 

### 4.4. Gas Chromatography-Mass Spectrometry (GC-MS)

For GC-MS analyses, an Agilent MSD quadrupole system (GC 7890A with MSD 5975C, Agilent Technologies, Waldbronn, Germany) coupled with a CIS 4C (cooled injection system) and an MPS 2 auto-sampler (Gerstel GmbH & Co. KG, Duisburg, Germany) were used. Data collection and analysis were performed with the MSD ChemStation E.02.00.493 (Agilent Technologies, Inc.) software. Quantification experiments were carried out on DB-FFAP and DB-5 capillaries (30 m length, 0.25 mm inner diameter, 0.25 µm film thickness, Agilent J&W GC Columns, USA), and for monitoring of the ratio of enantiomers the chiral capillaries Rt-bDEXsa, Rt-bDEXse, Rt-bDEXsm and Rt-yDEXsa (30 m length, 0.32 mm inner diameter, 0.25 µm film thickness, Restek GmbH, Bad Homburg, Germany) were utilised. Pre-columns of uncoated, deactivated fused silica (0.53 mm inner diameter, 0.5 to 3.5 m length) were used to ensure that no potentially present low-volatile residues could contaminate the analytical capillaries. The temperature programme of the column oven was as follows: 40 °C for 2 min, 4 °C/min up to 170 °C, 40 °C/min up to 230 °C (Rt-DEX capillaries), 240 °C (DB-FFAP) and 250 °C (DB-5), respectively, and hold time at end temperature of 5 to 10 min. As carrier gas, helium was used at a flow rate of 1.3 mL/min (velocity 41.1 cm/s). Injection of 1 or 2 µL was performed by the auto-sampler.

As transfer line into the MS, an uncoated, deactivated fused silica capillary (60 cm length, 0.25 mm inner diameter) was connected to the end of the analytical capillary and heated to 200 °C. Temperatures of the ion source and the quadrupole were 200 °C and 150 °C respectively. The CIS was programmed to the same temperatures as the column oven, so that cold-on-column injections were possible. Mass spectra were generated by electron ionisation (EI) at 70 eV ionisation energy and recorded in full scan mode (m/z range 40 to 250).

### 4.5. Determination of the Ratios of Enantiomers

For the separation of the pairs of enantiomers of the chiral metabolites of 1,8-cineole, four different capillaries (Rt-bDEXsa, Rt-bDEXse, Rt-bDEXsm, and Rt-yDEXsa) were evaluated for optimisation of the peak-to-peak resolution of the enantiomers by injecting a mixture of all the metabolites in a dichloromethane solution and by varying the heating rate. Besides the separation of each pair of enantiomers from each other, it was also important to confirm that no other metabolite coeluted with any one of the enantiomers, because the milk samples would most probably contain all metabolites. Moreover, the separation conditions for the mixture of reference substances were also tested on some human milk sample extracts and modified accordingly, where necessary, to avoid coelution with interfering matrix compounds. 

For calculation of the ratio of enantiomers (that is, the relative abundance of each enantiomer to the not separated mixture of enantiomers), the peak area of each enantiomer was divided by the sum of the peak areas of both enantiomers and multiplied by 100. With the known concentrations of the chiral metabolites in each milk sample, the absolute concentration of each single enantiomer was also calculated by multiplying the concentration with the respective percentage.

### 4.6. Quantification with Internal Standard and Matrix Calibration

Human milk samples were spiked with deuterium labelled 1,8-cineole (solution in dichloromethane) as internal standard prior to SAFE distillation. The amount of internal standard added was varied for each sample according to the sample volume and the subjective perception rating of the eucalyptus-like smell as a sign of the efficiency of transfer (~0.1–0.2 µg/g for samples with detectable odour and 100-fold less for odourless samples). Sample extracts were measured on both GC capillaries DB-FFAP and DB-5; thereby, the signal ratios of internal standard and analyte on DB-5 were used for quantification of 9-hydroxy-1,8-cineole while for all other metabolites the chromatograms on DB-FFAP were utilised. The peak areas of the metabolites were from extracted ion chromatograms of the m/z values given in Table 4 and the peak area of labelled 1,8-cineole was from the extracted ion chromatogram of m/z 157.

To set up the calibration curves, spiked matrix samples were used, taking into account the sample preparation. Therefore at first seven calibration spiking solutions in dichloromethane were prepared with descending concentrations of the following metabolites: 2,3-Dehydro-1,8-cineole, α2,3-epoxy-1,8-cineole, α2-hydroxy-1,8-cineole, β2-hydroxy-1,8-cineole, 4-hydroxy-1,8-cineole, 7-hydroxy-1,8-cineole and 9-hydroxy-1,8-cineole. 50 µL of each spiking solution containing all seven metabolites were added to ~50 mL human milk together with 35 µL of a solution of labelled 1,8-cineole before sample preparation via SAFE. This resulted in concentration ratios of metabolite to labelled 1,8-cineole of about 7:1, 3:1, 1:1, 0.6:1, 0.1:1, 0.05:1 and 0.01:1 for the respective calibration level (absolute amount of each metabolite in the milk samples between 65 and 0.02 µg; absolute amount of internal standard ~8 µg).

**Table 4:** Calibration curves used for quantification of metabolites of 1,8-cineole.

Metabolite	m/z for Data Analysis	Equation of Calibration Curve	Correlation Coefficient
2,3-dehydro-1,8-cineole (low range^a^)	109	y=6.5814x-0.0004	0.9988
a2,3-epoxy-1,8-cineole (low range^a^)	95	y=1.8892x-0.0002	0.9996
a2-hydroxy-1,8-cineole (high range^b^)	108	y=2.0255x-0.0069	0.9938
a2-hydroxy-1,8-cineole (low range^a^)	108	y=1.8600x-0.0007	0.9988
7-hydroxy-1,8-cineole (low range^a^)	111	y=0.2487x-0.0001	0.9926
9-hydroxy-1,8-cineole (low range^a^)	139	y=9.4586x-0.0001	0.9961
4-hydroxy-1,8-cineole (high range^b^)	112	y=2.1108x-0.0282	0.9996
4-hydroxy-1,8-cineole (low range^a^)	112	y=1.9539x-0.0010	0.9965
2-oxo-1,8-cineole (low range^a^)	82	y=4.5637x-0.0002	0.9995
3-oxo-1,8-cineole (high range^b^)	153	y=15.6904x-0.0002	0.9964
3-oxo-1,8-cineole (low range^a^)	153	y=15.9449x-0.0001	0.9995
a3-hydroxy-1,8-cineole (low range^a^)	108	y=15.2720x-0.0001	0.9962

^a^ concentration ratios of metabolite to labelled 1,8-cineole of about 1:1, 0.6:1, 0.1:1, 0.05:1, 0.01:1; ^b^ concentration ratios of metabolite to labelled 1,8-cineole of about 7:1, 3:1, 1:1, 0.6:1, 0.1:1.

The human milk used as calibration matrix was mixed from several smaller samples from different mothers to get a final volume of about 500 mL of which ~50 mL were taken for each calibration point. Additionally, ~50 mL were subjected to sample preparation without spiking with metabolites but only with the labelled 1,8-cineole as internal standard. This blank sample was used as a control for potential naturally occurring traces of metabolites. Altogether three mixed samples of human milk were each used for seven calibration points and the blank sample, resulting in three calibration samples for each level.

After measurement of the calibration samples, signal ratios of labelled 1,8-cineole to the respective metabolite were calculated from the respective chromatograms. Values of the blank sample were subtracted from those of the calibration samples. The signal ratios of the three calibration points of the same level were averaged and a calibration curve for each metabolite was calculated by linear regression.

For calculation of β2-hydroxy-1,8-cineole in the samples, the calibration curve of α2-hydroxy-1,8-cineole could be used because these metabolites are diastereomers and, thus, are expected to show equal behaviour during sample preparation and mass spectrometry and are differentiated only by their retention times on achiral GC-capillaries.

One sample had a very high ratio of α2-hydroxy-1,8-cineole to internal standard and thus the calibration range was extended for this sample with a calibration point at the exact ratio of 1:20.03 for metabolite to labelled 1,8-cineole (calibration sample spiked with 157.76 µg of α2-hydroxy-1,8-cineole and labelled 1,8-cineole as described above). This calibration point was also prepared in triplicate.

For α3-hydroxy-1,8-cineole and 3-oxo-1,8-cineole, reference substances were only available in very small amounts and in solutions with unknown concentration, thus no calibration could be undertaken as with the other metabolites. Nevertheless, the estimated concentrations of these substances were calculated in the analysed milk samples by using the calibration curves of the closely related metabolites α2-hydroxy-1,8-cineole and 2-oxo-1,8-cineole under the assumption of equal response in mass spectrometric detection. This assumption is only valid when comparing peak areas in total ion chromatograms because of the different fractionation patterns of each metabolite. Since the resolution was not sufficient in total ion chromatograms, all peak areas were determined from extracted ion chromatograms. Accordingly, these areas had to be mathematically converted to respective total ion chromatogram areas. This was realised by multiplication with a factor for the ratio of the total ion peak area, and the extracted ion peak area, determined from more than 20 independent measurements of the respective metabolite reference substance.

To improve linearity, the range of seven calibration points was divided into two ranges, including only the five highest and the five lowest calibration points respectively. For some metabolites, the calculated signal ratios of the samples were only in the range of the five lowest calibration points, and thus the respective calibration curve for the higher range is not reported.

Further optimisation of the calibration was carried out by varying several parameters and comparing the resulting calibration curves: Different weighting factors were tested, the area ratio was set alternatively as y-value or as x-value, the signals on DB-FFAP or on DB-5 were used and the worst matching calibration sample out of the three calibration points of the same level was excluded or left included. Finally, the best calibration equation for each metabolite was chosen based on the following criteria: With each calibration curve, concentration ratios were calculated from the measured signal ratio of the calibration samples and compared with the nominal concentration ratios. The calibration equation leading to the smallest deviation (*i.e.*, to the highest reovery rate) was chosen for final quantification of the human milk samples. The calibration equations are given in Table 4. 

## 5. Conclusions

Several human metabolites of the odorant 1,8-cineole were previously identified in human milk after an oral dose (100 mg) of the parent molecule 1,8-cineole [30]. Accordingly, the aim of this study was to determine which concentrations of each metabolite occurred in the breast milk. Measured concentrations varied over a range of some tens of µg/kg for most metabolites, both inter and intra-individually, but absolute concentrations were rather low over a range of up to 40 µg/kg milk. The only exception was the metabolite α2-hydroxy-1,8-cineole, which showed significantly higher concentrations of about 100–250 µg/kg and, thus, was clearly the main metabolite of 1,8-cineole in human milk, as has also been reported for blood plasma, urine [28,29,41], and human liver microsomes [27,28,54,55]. In some human milk samples, the concentration of this main metabolite was even higher than that of the remaining un-metabolised 1,8-cineole (as determined previously [39]). 

The total amount of metabolised 1,8-cineole in the samples increased with the time since the intake of the encapsulated 1,8-cineole, indicating that the metabolite concentrations in human milk were highly dynamic, probably due to transfer from the blood, or even to metabolic activity in the milk itself. It would be interesting to draw a conclusion about the percentage of 1,8-cineole that is converted to each metabolite. However, only roughly estimated mean values can be given because many parameters influence the concentration of metabolites in human milk, like general inter and intra-individual differences in metabolism and dependency of the metabolic rate on the amount of initially present 1,8-cineole. In total, 10%–70% (mean 30%–50%) of 1,8-cineole was metabolised, and the main metabolite α2-hydroxy-1,8-cineole accounted on average for about 70% of the metabolites, while the other metabolites were present in low but approximately equal amounts. 

Most of the metabolites were chiral, thus it was also interesting to determine the ratio of enantiomers, which potentially allows conclusions to be drawn about the enzymatic pathways involved. Here, large variations over the total sum of samples were also observed, but generally it can be said that the metabolites α2-hydroxy- and 3-oxo-1,8-cineole were nearly enantiomerically pure in human milk, while all other metabolites of the present study showed enantiomeric ratios of about 30:70 to 80:20.

Finally, it seems justifiable to say that the present study was a big step towards the characterisation of the human metabolism of 1,8-cineole. While former studies on blood and urine [28,29] did not provide such detailed analyses on metabolism, which was probably also due to lower doses of 1,8-cineole applied in these studies, it is now also possible to compare the presented results for human milk with a very recent study [41] on human urine using the same dosage in the form of a Soledum® capsule. This comparison showed that there were noteworthy differences in the overall metabolites identified and in the enantiomeric composition of the single substances. These observations confirm the theory that metabolite profiles in (human) milk might be substantially different from those in the commonly analysed body fluids of blood and urine and are thus worth analyzing, especially in the context of breastfeeding and the uptake of potentially harmful substances by the nursling. Thus, further studies are needed on this topic in order to establish reliable relationships between the metabolic situation in human milk and the other body compartments.

## Supplementary Files

Supplementary File 1 Supplementary material (PDF, 15 KB)
