# Supplementary material for: Characterisation of the Metabolites of 1,8-Cineole Transferred into Human Milk: Concentrations and Ratio of Enantiomers
Source: Metabolites. 2013 Jan 30;3(1):47–71. doi: 10.3390/metabo3010047 (PMC3901259; doi:10.3390/metabo3010047)
Supplement: Supplementary File 1 — Supplementary material (PDF, 15 KB) [file metabolites-03-00047-s001.pdf]

Supplementary material

Table S1. Chiral GC capillaries suitable for separation of enantiomers of 1,8-cineole metabolites and respective retention indices.

**metabolite**	**chiral GC capillary for separation of enantiomers**	**retention index of the first eluting enantiomer**	**retention index of the second eluting enantiomer**
2,3-dehydro-1,8-cineole	Rt-bDEXsm	1058	1066
α2,3-epoxy-1,8-cineole	Rt-bDEXsa	1333	1336
	Rt-bDEXse	1199	1210
	Rt-bDEXsm	1264	1277
	Rt-yDEXsa	1339	1342
α2-hydroxy-1,8-cineole	Rt-bDEXsa	1484	1487
	Rt-yDEXsa	1489	1493
β2-hydroxy-1,8-cineole	Rt-bDEXsa	1446	1452
	Rt-bDEXsm	1330	1334
	Rt-yDEXsa	1452	1458
α3-hydroxy-1,8-cineole	Rt-bDEXsa	1511	1517
	Rt-yDEXsa	1517	1524
9-hydroxy-1,8-cineole	Rt-bDEXsm	1404	1419
2-oxo-1,8-cineole	Rt-bDEXsa	1501	1534
	Rt-bDEXse	1292	1314
	Rt-bDEXsm	1315	1345
	Rt-yDEXsa	1510	1551
3-oxo-1,8-cineole	Rt-bDEXsa	1425	1443
	Rt-bDEXse	1267	1257
	Rt-bDEXsm	1290	1312
	Rt-yDEXsa	1426	1448
